# The Emerging Role of MTHFD Family Genes in Regulating the Tumor Immunity of Oral Squamous Cell Carcinoma

**DOI:** 10.1155/2022/4867730

**Published:** 2022-06-03

**Authors:** Wei Wang, Wenli Gu, Hai Tang, Zhaoyi Mai, Hui Xiao, Jianjiang Zhao, Jiusong Han

**Affiliations:** ^1^Stomatological Hospital, Southern Medical University, Guangzhou 510280, China; ^2^Shenzhen Stomatological Hospital, Southern Medical University, Shenzhen 518001, China

## Abstract

**Objective:**

To investigate the function and regulatory mechanisms of methylenetetrahydrofolate dehydrogenase (MTHFD) family genes in oral squamous cell carcinoma (OSCC), especially focus on their regulating role in tumor immunity.

**Methods:**

The publicly available data from the TCGA database were used to investigate the expression pattern and regulatory role of MTHFD family genes in OSCC. More importantly, the involvement of MTHFD family genes in tumor immunity was investigated in terms of immune and stromal cell infiltration in tumor microenvironment, tumor-infiltrating immune cells, and immunomodulatory genes (e.g., immunoinhibitory genes and immunostimulatory genes). Statistical analysis was performed using R software packages and public web servers.

**Results:**

MTHFD family genes were considerably upregulated in OSCC as compared with normal oral tissue. Patients with high MTHFD2 expression presented worse survival outcomes than those with low MTHFD2 expression. Functional enrichment analysis showed that the top 100 positively and negatively correlated genes of the MTHFD family genes were significantly enriched in several KEGG pathways, including cell cycle, spliceosome, DNA replication, and Th17 cell differentiation. As a result of tumor immunity analysis, MTHFD2L expression was found to be negatively related to the Estimate-Stromal-Immune score in OSCC; however, there was no statistical significance between the Estimate-Stromal-Immune score and MTHFD1, MTHFD1L, or MTHFD2 in OSCC. Additionally, MTHFD family genes were found to be significantly positively correlated with tumor-infiltrating immune cells, including Treg and Th17 cells. Moreover, MTHFD family genes were significantly correlated with several immune inhibitory genes such as CD274 and CTLA4 and several immune-stimulatory genes such as CXCL12, CXCR4, and TMIGD2.

**Conclusion:**

Given the expression pattern, prognostic value, biological functions, and involvement in tumor immunity, MTHFD family genes could serve as potential therapeutic biomarkers in targeting tumor immunity in oral cancer.

## 1. Introduction

Oral squamous cell carcinoma (OSCC) is one of the most common malignancies in the head and neck region. Common risk factors include smoking, consuming alcohol, and chewing betel nuts [1]. Current treatments for OSCC include radical surgical resection with reconstruction, chemotherapy, radiotherapy, and immunotherapy. Although strategies for treating OSCC have improved in recent decades, the prognosis of OSCC remains low, with a long-term disease-free survival rate of around 50% [2]. Thus, accurate prediction of OSCC prognosis is essential to successful clinical management and individualized treatment. The tumor-node-metastasis (TNM) staging system for OSCC remains the primary prognostic indicator in clinical practice. However, the OSCC patients with the same TNM stage might have significantly different clinical outcomes. Therefore, it is imperative to identify novel and robust prognosis and predictive biomarkers to improve the prognosis of OSCC.

Folate metabolism, known as one-carbon (1C), supplies a one-carbon (1C) group for a wide range of transformations and supports multiple physiological processes. These include purines and thymidine biosynthesis, amino acid homeostasis (glycine, serine, and methionine), redox homeostasis, and epigenetic maintenance [3]. Recent studies have shown that the mitochondrial one-carbon pathway is often reprogrammed in cancer cells. One-carbon metabolism generates biosynthetic substrates that are necessary for the growth and survival of proliferating cells [4]. Inhibition of folate metabolism blocks cellular proliferation [5]. Furthermore, folate-metabolizing enzymes are essential regulators that directly control tumor metabolic balance and are highly correlated with cancer malignancy [6].

The methylenetetrahydrofolate dehydrogenase (MTHFD) family genes are essential mitochondrial folate metabolism-regulating enzymes that play a crucial role in cell nucleic acid synthesis and oxidative stress. MTHFD family genes include MTHFD1, MTHFD1L, MTHFD2, and MTHFD2L. MTHFD2 and MTHFD2L catalyze the conversion of 5,10-MeTHF to 10-formyl-THF in the mitochondria, while MTHFD1L converts 10-formyl-THF to formate and carry it out of the mitochondria [3]. Formate is catalyzed in the cytoplasm to create 10-formyl-THF and 5,10-MeTHF, which are involved in cell metabolism. MTHFD2, MTHFD2L, and MTHFD1L catalyze serine catabolism, which provides the principal supply of one-carbon units and glycine for cell biosynthesis and maintains systemic metabolism homeostasis [3]. Recent studies have shown that the MTHFD family genes are upregulated in cancer. MTHFD1 was overexpressed in hepatocellular carcinoma and associated with a poor prognosis [7]. MTHFD1L was highly expressed in tongue squamous cell carcinoma, colorectal cancer, bladder cancer, and osteosarcoma and associated with poor disease prognosis. MTHFD1L also plays an essential role in cell proliferation and invasion [8–10]. MTHFD2 expression was markedly elevated in both solid and hematological malignancies. MTHFD2 could confer redox homeostasis, promote cancer cell growth metastasis, and correlate with poor survival [11–13]. Although the alterations in MTHFD family genes are associated with cancer progression, their precise roles in the development of OSCC remain unknown. Elucidating the relationship between MTHFD family genes and OSCC progression might provide meaningful guidance for improving the therapeutic outcome.

Therefore, this study aimed to explore the prognostic values of MTHFD family genes in OSCC and to estimate the association between MTHFD family genes and tumor immunity. Our work indicates that MTHFD family genes have the potential to serve as biomarkers for OSCC diagnosis and prognosis.

## 2. Materials and Methods

### 2.1. Expression of MTHFD Family Genes in OSCC and Pan-Cancer

This study was designed as reported by other groups [14–16]. The cBioPortal for Cancer Genomics was used to visualize the three-dimensional (3D) protein structure of MTHFD family genes. Data were obtained from UCSC XENA (https://xenabrowser.net/datapages/) and are RNAseq data in TPM format for The Cancer Genome Atlas (TCGA) and Genotype-Tissue Expression (GTEx) processed in a uniform manner by the Toil process. We analyzed the mRNA expression levels of MTHFD family genes in 33 different tumor types: adrenocortical carcinoma (ACC), bladder urothelial carcinoma (BLCA), breast cancer (BRCA), cervical squamous cell carcinoma and endocervical adenocarcinoma (CESC), cholangiocarcinoma (CHOL), colon adenocarcinoma (COAD), lymphoid neoplasm diffuse large B-cell lymphoma (DLBC), esophageal carcinoma (ESCA), glioblastoma multiforme (GBM), head and neck squamous carcinoma (HNSC), kidney chromophobe (KICH), kidney renal clear cell carcinoma (KIRC), kidney renal papillary cell carcinoma (KIRP), acute myeloid leukemia (LAML), brain lower grade glioma (LGG), liver hepatocellular carcinoma (LIHC), lung adenocarcinoma (LUAD), lung squamous cell carcinoma (LUSC), mesothelioma (MESO), ovarian serous cystadenocarcinoma (OV), pancreatic adenocarcinoma (PAAD), pheochromocytoma and paraganglioma (PCPG), prostate adenocarcinoma (PRAD), rectum adenocarcinoma (READ), sarcoma (SARC), skin cutaneous melanoma (SKCM), stomach adenocarcinoma (STAD), testicular germ cell tumors (TGCT), thyroid carcinoma (THCA), thymoma (THYM), uterine corpus endometrial carcinoma (UCEC), uterine carcinosarcoma (UCS), and uveal melanoma (UVM). RNAseq data in TPM format were log2-transformed for analysis and comparison. The Mann–Whitney U method was used for testing. The ggplot2 in the R package was used to visualize the results.

We further analyzed the mRNA expression levels of MTHFD family genes in 329 OSCC cancer and 32 adjacent normal samples. The data was normalized by transforming the RNA-sequencing data from FPKM (fragments per kilobase per million) format to TPM (transcripts per million reads) and then converted by log2 transformed. The ggplot tool in R was used to visualize the expressions of these genes (version 3.3.3). The expressions of MTHFD family genes in 32 pairs of tumor samples and their matched adjacent normal samples were analyzed by Student's *t*-test, while the Mann–Whitney *U* test analyzed the unpaired samples. Receiver operating characteristic (ROC) curve analysis was performed to evaluate the sensitivity and specificity of the MTHFD family genes for OSCC diagnosis using the R package. An area under the curve (AUC) value was calculated and used to evaluate the ROC effect. The abscissa indicates the false positive rate (FPR), and the ordinate represents the true positive rate (true positive rate, TPR) in ROC curves.

### 2.2. Analysis of Immunohistochemistry

Four primary OSCC tissues and adjacent normal specimens were obtained from patients who underwent surgical resection at the Department of Craniofacial Surgical Resection, Stomatological Hospital, Southern Medical University, Guangzhou, China. All patients provided written informed consent prior to sample collection. The study protocol was approved by the Ethics Committee of Stomatological Hospital, Southern Medical University. The harvested specimens were fixed with 4% paraformaldehyde (PFA) and sectioned at 4 *μ*m thickness. Then, deparaffinized sections were soaked in a 3% H_2_O_2_ for 10 min and blocked with fetal bovine serum for 30 min. After that, sections were incubated with a polyclonal rabbit anti-human MTHFD2 antibody (1 : 200, NBP1-33200, Novus) at 4°C overnight, followed by a horseradish peroxidase-conjugated goat anti-rabbit secondary antibody (1 : 10 000, GK600705A, Gene Tech) for 1 h at room temperature. Finally, slides were treated with 3,3-diaminobenzidine (DAB, ZLI-9018, OriGene) for 2 min and counterstained with hematoxylin dye for 1 min at room temperature. Stained sections were evaluated using a light microscope (Olympus, Japan) at a magnification of ×200.

### 2.3. Association between MTHFD Family Genes and Clinical Characteristics

The binary logistic model was used to evaluate the connection between MTHFD family gene expression and clinical characteristics (Table 1). The statistical approach of logistic regression was used to forecast the relationship between predictors and predicted variables. The independent variable, each MTHFD family gene, was classified into the low expression group and high expression group. The dependent variable characteristics were also divided into two different categories, for example, T stage (higher T stage (T3 and T4) versus lower T stage (T1 and T2), age (>60 versus ≤ 60), and alcohol history (Yes versus No). The data for OSCC were obtained from the HNSCC sample in the TCGA database. The OSCC samples without clinical information were removed. The relationship between the expression level of each MTHFD family gene and clinical stages was analyzed by ggplot package in R program and visualized by box plots. To determine the association between each pair of MTHFD family genes, a Pearson correlation coefficient (PCC) study was conducted. The expression level of each MTHFD gene was determined in TCGA-HNSCC tumor samples. Between each pair, the *r* and *P* values were determined. Following that, a heatmap was created using the ggplot2 package (version 3.3.3) in the R software (version 3.6.3).

### 2.4. Prognostic Value Estimation of MTHFD Family Genes in OSCC

Based on RNA-sequencing expression data of 9736 tumors and 8587 normal samples from the TCGA and GTEx projects, given a list of custom cancer types, GEPIA2 (accessed on March 20, 2021) (URL: http://gepia2.cancer-pku.cn/) would provide a survival heatmap to show the survival analysis results of gene lists based on multiple cancer types. In the Kaplan–Meier analysis, overall survival (OS), disease-specific survival (DSS), and progression-free survival (PFI) were chosen as predictive factors. Cox regression studies were performed using the coxph function from the survival package of R (version 3.6.3) along with the Cox regression module. Using the TCGA-OSCC dataset, we made a predictive nomogram, allowing doctors to forecast the likelihood of individual patient mortality and guide patient evaluation and treatment decision-making. Overall survival was selected as the prognostic outcome type. The calibration plot was created and visualized using the rms package (version 6.2–0) and survival package in the R program. There are four lines in the calibration plot representing the 1-, 3-, and 5-years predicted survival model, the actual situation, and the ideal line (diagonal line, grey).

### 2.5. Identification of Correlated Genes with MTHFD Family Genes

The MTHFD family genes-based gene-gene interaction (GGI) network was built using the GeneMANIA website (URL: http://genemania.org). All 4 MTHFD family genes were used as the input, and the top few functions with the lowest FDR values were shown in the network. The GGI network was constructed by an automatically selected weighting method. The expression pattern of correlated genes with MTHFD family genes in OSCC samples was shown using a heatmap created by the R tool ggplot2 (version 3.3.3). NetworkAnalyst is a visual analytics platform for comprehensive gene expression profiling and meta-analysis (URL: https://www.networkanalyst.ca/). Firstly, the organism was designated as *H. sapiens* (human), and all MTHFD family genes were uploaded by using the following Entrez IDs: 4522, 25902, 10797, and 441024. Secondly, based on the STRING interactome database, generic protein-protein interactions (PPI) were chosen to be constructed. All interactions required experimental evidence, and the minimum required interaction score was 0.40 (medium confidence). The confidence score cutoff was set at 900. Afterward, a network including 12 nodes, 16 edges, and 3 seeds was constructed and viewed.

### 2.6. Identification of the Functional Terms of the Correlated Genes of MTHFD Family Genes

The top 100 positively and top 100 negatively correlated genes of MTHFD family genes were analyzed using functional enrichment analysis to identify significantly enriched functional terms. GO keywords such as cellular component, biological process, and molecular function, as well as KEGG pathways significantly enriched by the associated genes, were detected using a criterion of *P* < 0.05. Only the top 30 terms listed by ascending order of the *P* value were collected to produce a bubble chart if there were more than 30 terms that were substantially enriched at this threshold setting; otherwise, all of the terms were utilized. The R package “ggplot2” was used to generate bubble charts to show the enrichment findings. In addition, the gene set enrichment analysis (GSEA) was performed to verify the results of the GO and KEGG analysis. The TCGA-OSCC data collection was consulted to determine the differentially expressed genes (DEGs) that were substantially dysregulated across OSCC samples and healthy control samples by applying the R package “DESeq2” (1.26.0). The experimental group was made up of clinical status-tumor samples, while the reference group for differential expression analysis was composed of clinically healthy control samples. The most significantly correlated genes with MTHFD genes (*P* value < 0.05 and |cor Pearson (*r*)| > 0.4) were used as the input genes for performing GESA analysis. According to such selection criteria, 3609 genes correlated with MTHFD1, 576 genes correlated with MTHFD1L, 3270 genes correlated with MTHFD2, and 85 genes correlated with MTHFD2L were obtained. After integrating these genes and removing the repeated genes, 5558 genes were obtained and used as the input for carrying out GSEA analyses. The log_2_FC values of these MTHFD genes-correlated genes were obtained. The clusterProfiler package in R was used to conduct the GSEA analysis. Three databases were used to obtain the pathways: the KEGG pathway database, the WikiPathways (WP) database, and the Reactome (REAC) database. Significantly enriched terms were defined as functional terms fulfilling the conditions of *P* adjust <0.05, |NES| > 1, and FDR (also known as q-val) < 0.25.

### 2.7. The Correlation between MTHFD Family Genes and Immunity in OSCC

The ESTIMATE (estimation of stromal and immune cells in malignant tumor tissues using expression data) algorithm was applied to estimate the stromal and immune scores of a series of OSCC cancer tissues based on their transcriptional profiles. The correlation between MTFHD family genes and Estimate-Stromal-Immune score was estimated by the estimate package (version 1.0.13) in the R program (version 3.6.3). The correlation of each MTHFD family gene with tumor immune infiltration cells (TIICs) in OSCC samples was evaluated by the Pearson statistical approach using the “GSVA” package (version 1.34.0) in R (version 3.6.3) and illustrated using a lollipop plot. Using the ggplot package in the R software, we presented the relationship between MTHFD family genes and immune inhibitory/stimulatory genes using a correlation heatmap approach.

## 3. Results

### 3.1. The Expression Pattern of MTHFD Family Genes in OSCC

**Figure 1:**
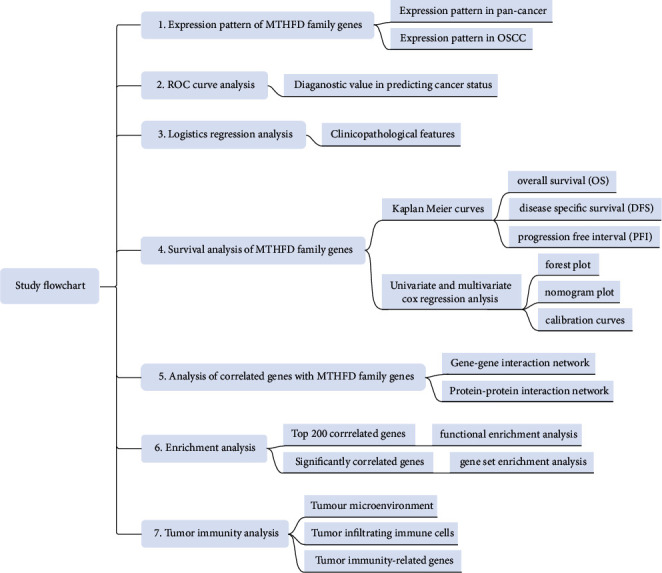
The study flowchart of the current research.

**Figure 2:**
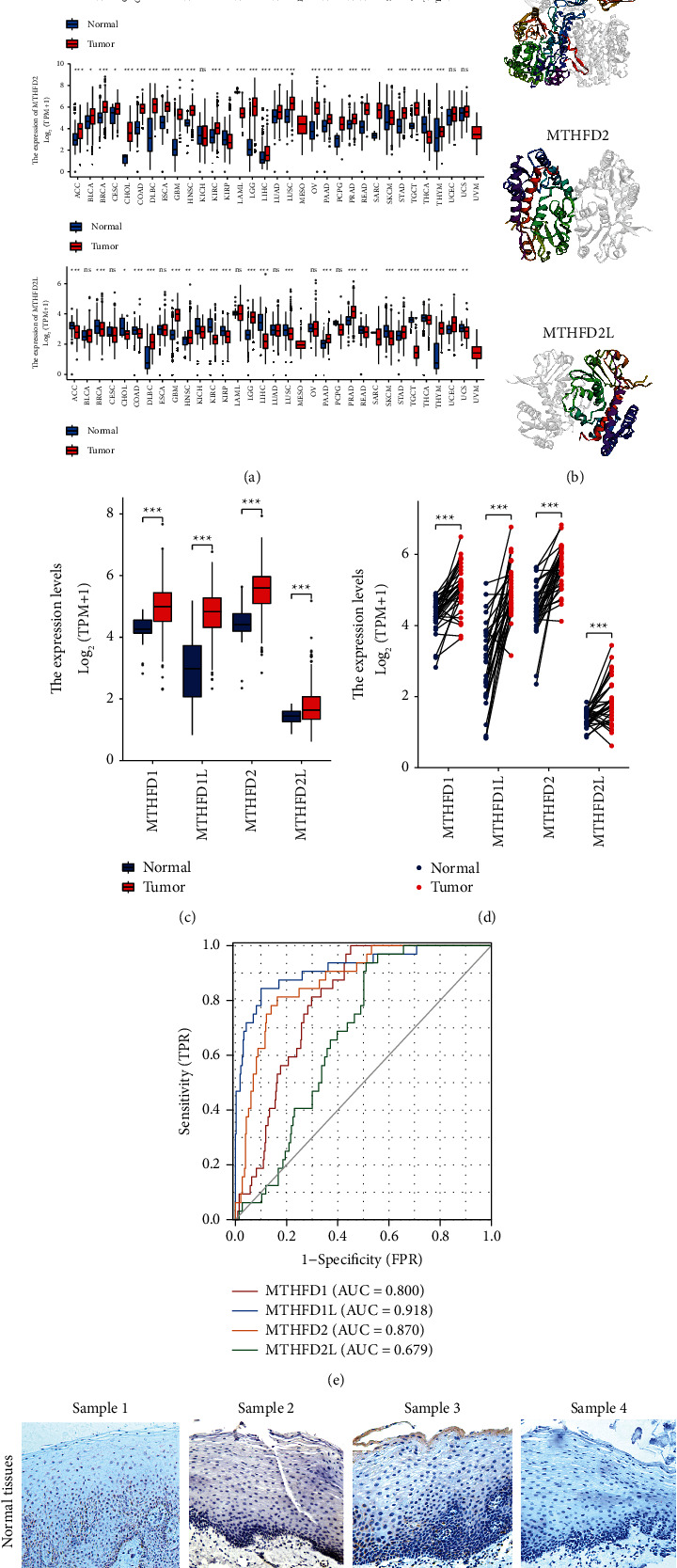
The expression pattern of MTHFD family genes. (a) The expression pattern of each MTHFD family gene in pan-cancer. (b) The 3D structure of MTHFD family genes. (c) The expression pattern of MTHFD family genes in OSCC by unpaired samples analysis. (d) The expression pattern of MTHFD family genes in OSCC by paired samples analysis. (e) ROC curves for evaluating the diagnostic value of MTHFD family genes for OSCC. (f) Immunohistochemical expression of MTHFD2 in OSCC tissue and adjacent normal tissues (×200).

The study strategy of the present study is illustrated in Figure 1.  Figure 2(a) shows the expression pattern of each MTHFD family gene in the pan-cancer. MTHFD1, MTHFD1L, and MTHFD2 have been found significantly upregulated in a variety of cancers (e.g., BLCA, BRCA, CESC, COAD, DLBC, ESCA, GBM, HNSC, KIRP, LGG, LUSC, PAAD, PRAD, READ, SKCM, STAD, TGCT, THCA, THYM, UCEC, and UCS). However, MTHFD1 was downregulated in CHOL, KICH, and LIHC. MTHFD1L was downregulated in ACC and KICH. MTHFD2 was downregulated in KIRP and THCA. MTHFD2L was found to be significantly upregulated in several cancer types (e.g., DLBC, GBM, HNSC, LGG, PAAD, PRAD, STAD, THYM, and UCEC) and downregulated in many cancer types (e.g., ACC, BRCA, CHOL, COAD, KICH, KIRC, KIRP, LIHC, LUSC, READ, SKCM, TGCT, THCA, and UCS).  Figure 2(b) shows the 3D protein structure of four MTHFD family genes. The mRNA expression of the MTHFD family genes was significantly upregulated in OSCC samples as compared with normal oral samples in both unpaired and paired sample analysis (*P* < 0.001) (Figure 2(c) and 2(d)). The ROC curve was used to assess the diagnostic utility of MTHFD family genes. The variables MTHFD1, MTHFD1L, and MTHFD2 showed a high predictive accuracy to identify OSCC samples from normal controls (AUC = 0.800, 0.918, 0.870, resp.), while the variable MTHFD2L had a lower predictive accuracy (AUC = 0.679) (Figure 2(e)). Immunohistochemical staining of four pairs of clinical OSCC samples confirmed that the level of MTHFD2 in tumor tissues was higher than that in adjacent normal tissues (Figure 2(f)).

### 3.2. Associations between MTHFD Family Genes Expression and Clinicopathologic Characteristics in OSCC

The correlation between the mRNA expression of MTHFD family genes and clinical characteristics in OSCC patients was explored by logistic regression analysis (Table 1). MTHFD1L expression was positively associated with histologic grade (*P*=0.027). MTHFD2 expression was significantly positively linked with T stage (*P*=0.018) and clinical stage (*P*=0.028). MTHFD2L expression was positively correlated with gender (*P*=0.030) and negatively correlated with age (*P*=0.027).

### 3.3. Value of MTHFD Family Genes in Predicting Prognosis in OSCC

**Figure 3:**
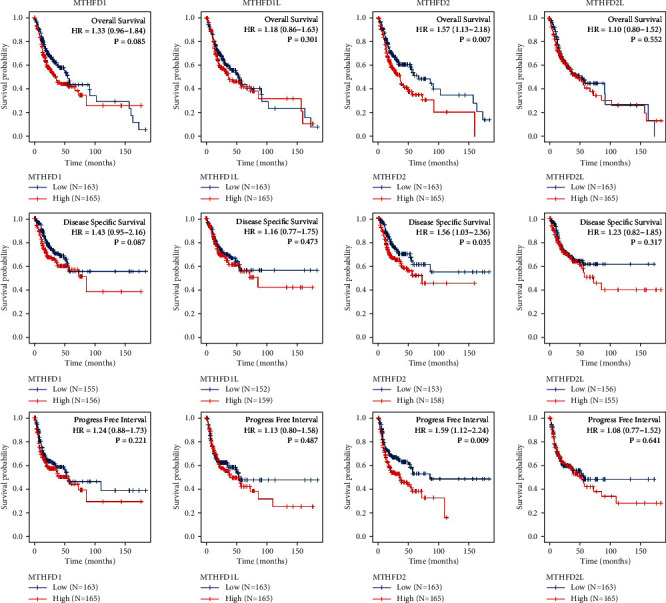
The Kaplan–Meier curves showed the relationship between MTHFD family genes and three types of prognostic outcomes (overall survival (OS), disease-specific survival (DSS), and progression-free survival (PFI)) in OSCC. Patients were divided into two groups based on the median of gene expression. The survival outcomes of the two groups were evaluated by log rank tests. *P* < 0.05 was statistically significant.

**Figure 4:**
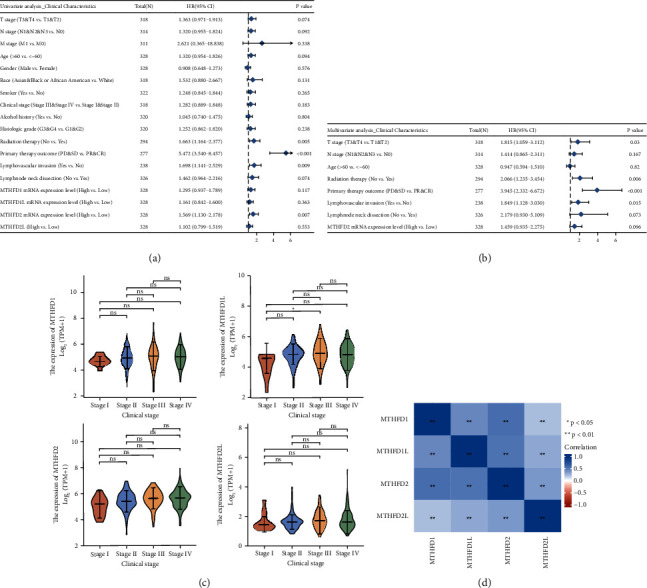
The relationship between each MTHFD family gene and clinical characteristics. (a, b) Univariate and multivariate Cox regression analyses of the association between MTHFD family genes and clinical characteristics. (c) The relationship between each MTHFD family gene and pathologic stages of OSCC. (d) Heatmap of genetic correlation between the expression patterns of MTHFD family genes in OSCC.

**Figure 5:**
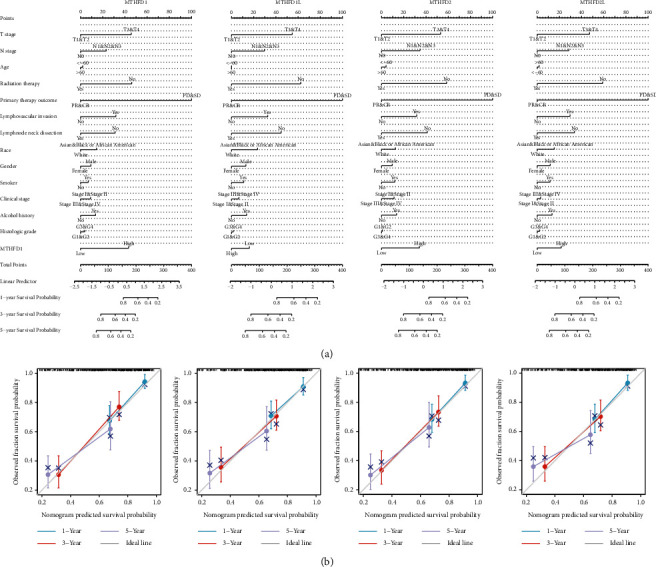
The nomogram plots and calibration plots. (a) The nomogram plots were used for predicting the survival probability of OSCC at 1-, 3-, and 5-years, integrating various clinicopathological factors and MTHFD family genes. (b) The calibration plots were used to evaluate the accuracy of the model established in the nomogram plot.

The Kaplan–Meier curves showed that OSCC patients with higher MTHFD2 expression showed a lower OS (*P* = 0.007), a poorer disease-specific survival (DSS) (*P* = 0.035), and a lower progression-free interval (PFI) (*P* = 0.009) (Figure 3). Overexpression of MTHFD2 was associated with a poorer prognosis of OSCC. However, the expression of MTHFD1, MTHFD1L, and MTHFD2L was not correlated with OS, DSS, and PFI. The findings of univariate and multivariate Cox regression studies are shown in Figure 4(a) and 4(b). The result revealed that primary therapy outcome was an independent factor for prognosis prediction (*P* < 0.001). As shown in Figure 4(c), the expression of MTHFD1L in stage III OSCC patients was significantly higher than that in stage I OSCC patients (*P* adjust = 0.027). However, there were no significant associations between pathologic stages and the expression of MTHFD1, MTHFD2, and MTHFD2L in TCGA-OSCC samples (*P* > 0.05). There was a significantly positive correlation between each pair of MTHFD family genes (*P* < 0.01). The correlation was most significant between MTHFD1 and MTHFD2 (*r* = 0.555) (Figure 4(d)). The nomogram was constructed to visualize the relationship between MTHFD family genes and survival probability. Patients with a higher number of total points had poorer survival outcomes. Calibration curves showed that MTHFD family genes had good predictive power for 1-year and 3-year overall survival (Figure 5).

### 3.4. The Coexpressed and Correlated Genes of MTHFD Family Genes

**Figure 6:**
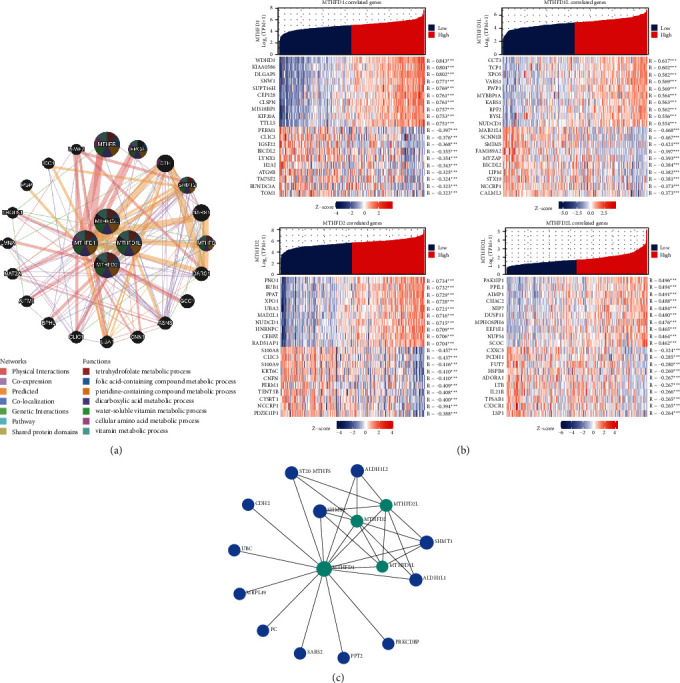
The coexpressed and correlated genes of the MTHFD family genes. (a) The gene-gene interaction network showed the top 20 coexpressed genes of MTHFD family genes. (b) The heatmap showed the expression pattern of the top 10 positively and top 10 negatively correlated genes of each MTHFD family gene. (c) The protein-protein interaction network showed the 12 interacted genes of MTHFD family genes.

**Figure 7:**
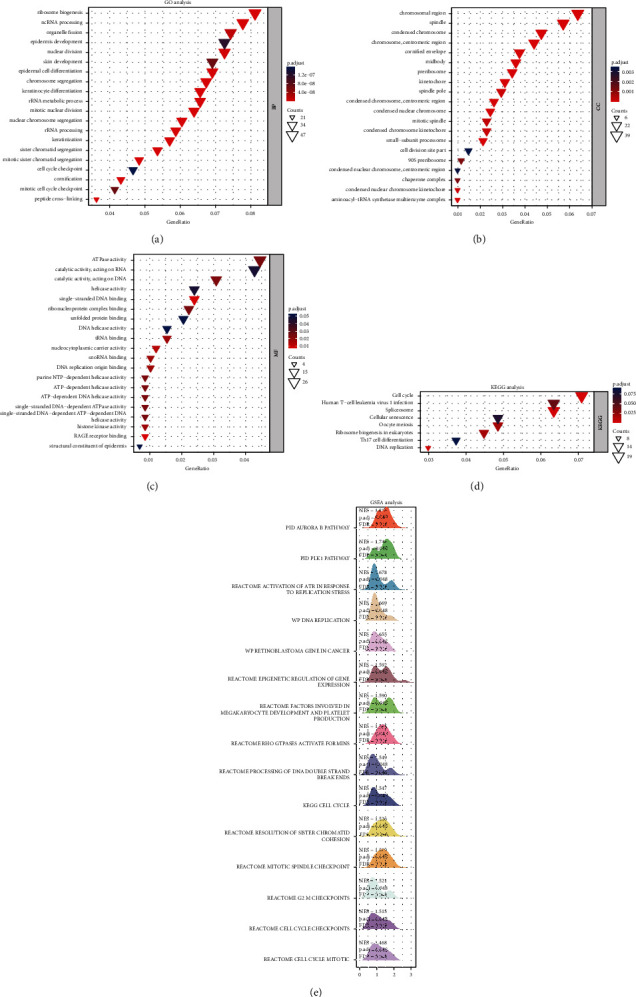
Functional enrichment analysis and gene set enrichment analysis of the correlated genes of MTHFD family genes in OSCC. (a)–(d) GO and KEGG analysis showed the top 20 functional terms enriched by the top 100 positively and top 100 negatively correlated MTHFD family genes. (e) The mountain plot showed the results of gene set enrichment analysis (GSEA), which was based on the significantly correlated genes (correlation coefficient value |r| > 0.4; *P* < 0.05) of MTHFD family genes.

Based on the STRING database, a GGI network of MTHFD family genes was constructed. The top 20 coexpressed genes were selected as essential hub node genes with high connectivity (Figure 6(a)). These genes, including MTHFR, SHMT2, and MTHFS, were involved in critical biological functions such as tetrahydrofolate metabolism, folic acid metabolism, pteridine metabolism, and amino acid metabolism. Then, a heatmap was used to depict the expression pattern of the top ten positively correlated genes of the MTHFD family genes, including WDHD1, CCT3, PNO1, and P1K1IP1, and the top ten negatively expressed genes, including CLIC3, PERM1, S100A8, and CXXC5 (Figure 6(b)). PPI network indicated that 12 genes (CDH2, UBC, MRPL49, PC, SARS2, PPT2, PRKCDBP, ALDH1L1, ALDH1L2, SHMT1, SHMT2, and ST20-MTHFS) were significantly associated with the MTHFD family genes expression (Figure 6(c)). Moreover, we uploaded the selected top 100 positively and 100 negatively correlated genes of MTHFD family genes to conduct GO and KEGG gene enrichment analysis. The top 20 functional terms are presented in Figure 7(a). GO gene enrichment analysis indicated these correlated genes were mainly enriched in ribosome biogenesis, ncRNA processing, organelle fission, nuclear division, chromosome segregation, and cell cycle checkpoint. The KEGG enrichment analysis showed that these correlated genes were predominantly associated with cell cycle, human T-cell leukemia virus 1 infection, spliceosome, cellular senescence, oocyte meiosis, ribosome biogenesis in eukaryotes, Th17 cell differentiation, and DNA replication (Figure 7(a)). The GSEA findings revealed that these correlated genes were considerably enriched in numerous signaling pathways, including cell cycle-related pathways (cell cycle, cell cycle mitotic, cell cycle checkpoints, and G2 M checkpoint), PLK1 pathway, DNA replication pathway, and Aurora B pathway (Figure 7(b)).

### 3.5. The Associations between MTHFD Family Genes and Tumor Immunity in OSCC

**Figure 8:**
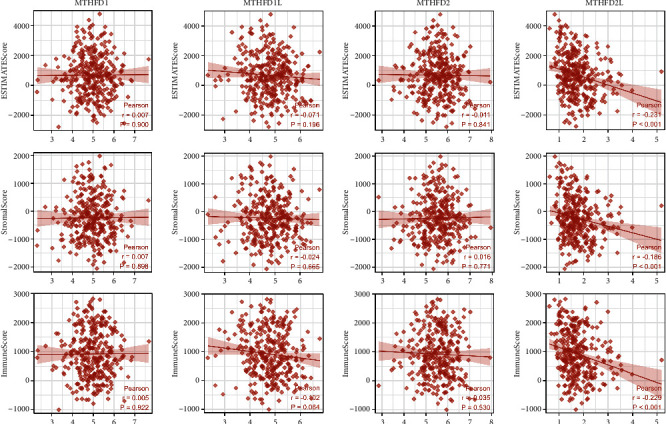
The correlation between MTHFD family genes and tumor microenvironment by investigating the Estimate-Immune-Stromal score in OSCC.

**Figure 9:**
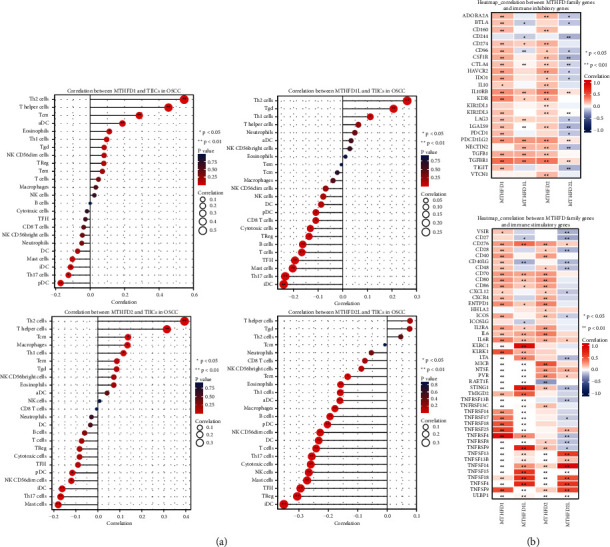
The association of MTHFD family genes and tumor immunity in OSCC. (a) Correlation between MTHFD family genes and immune cell infiltration in OSCC. (b) Correlation between MTHFD family genes and immune inhibitory/stimulatory genes in OSCC.

To further explore the relationships between the MTHFD family gene expression and immunity, three aspects were analyzed, including tumor microenvironment, tumor immune infiltration cells, and immune-related genes. We assessed the correlations between the MTHFD family genes and tumor microenvironment using the ESTIMATE algorithm, which determined the stromal cell and immune cell indexes present in tumor tissues. Our study demonstrated that the MTHFD2L expression was adversely linked with the ESTIMATEScore (*r* = −0.231; *P* < 0.001), the StromalScore (*r* = −0.186; *P* < 0.001), and the ImmuneScore (*r* = −0.229; *P* < 0.001) (Figure 8). This result suggested that MTHFD2L was positively correlated with the tumor purity of OSCC. However, there was no statistical significance between MTHFD1, MTHFD1L, or MTHFD2 and the tumor microenvironment in OSCC. The Pearson correlation analysis revealed that MTHFD family genes were significantly associated with immune cell infiltration, such as Treg and Th17 cells (Figure 9(a)). Furthermore, there are 24 immune inhibitory genes correlated with MTHFD family genes, including CD274 and CTLA-4, and 45 immune-stimulatory genes such as CXCL12, CXCR4, and TMIGD2 (Figure 9(b)).

## 4. Discussion

OSCC was one of the most common epithelial malignancies with a poor 5-year survival rate. At present, the most common strategies to diagnose OSCC are the comprehensive clinical examination and histological analysis of the suspicious area. However, early diagnosis is still difficult due to the limited sensitivity and specificity. There were also no accurate strategies to predict the prognosis of OSCC. Thus, it was urgent to identify novel and effective biomarkers for OSCC diagnosis and prognosis and to further explore its related mechanisms.

Our study revealed that MTHFD family genes were significantly upregulated in OSCC compared with normal oral tissue. The overexpression of MTHFD2 was associated with worse prognosis in OSCC. Several KEGG pathways were strongly associated with MTHFD family genes, such as cell cycle, human T-cell leukemia virus 1 infection, spliceosome, cellular senescence, oocyte meiosis, ribosome biogenesis in eukaryotes, Th17 cell differentiation, and DNA replication. MTHFD family genes were coexpressed with several important genes, such as SHMT2 and MTHFR. Additionally, MTHFD family genes were found to be significantly positively correlated with tumor-infiltrating immune cells, including Treg and Th17 cells. Furthermore, MTHFD family genes were significantly correlated with immunoinhibitory genes (e.g., CD274 and CTLA4) and immunostimulatory genes (e.g., CXCL12, CXCR4, and TMIGD2). These findings offer insights into the current understanding of MTHFD family gene function in OSCC.

A few previously published studies had reported that MTHFD family genes were upregulated in a variety of malignancies, such as acute leukemia, bladder cancer, and colorectal cancer [8, 12, 17, 18]. In line with these studies, our study showed that the mRNA expressions of MTHFD family genes were significantly upregulated in OSCC samples as compared with healthy oral samples using both unpaired and paired sample analysis. Furthermore, MTHFD family genes are closely related to the prognosis of many cancers. MTHFD1 expression was associated with a worse prognosis in acute leukemia and hepatocellular cancer patients [7, 17]. Patients with increased MTHFD1L expression had a poorer survival rate in both colorectal cancer (CRC) and hepatocellular carcinoma [18]. MTHFD2 overexpression is also linked with a poor clinical outcome in several cancers [11, 12, 19, 20]. Knockdown of MTHFD2 in breast cancer cells reduced tumor growth and affected many important metabolic pathways, indicating that MTHFD2 might be a central metabolic enzyme in cancer cells [21].

However, the functional role of MTHFD family genes in OSCC diagnosis and prognosis is unclear. In the present study, we showed that MTHFD1, MTHFD1L, and MTHFD2 had a high accuracy to differentiate OSCC tissue from normal tissue. Survival analysis revealed that MTHFD family genes had good predictive power for 1-year and 3-year overall survival of OSCC patients, indicating that MTHFD family genes could be used as an independent risk factor for judging the prognosis of OSCC patients. Further studies are warranted to validate this finding in a large number cohort.

The GGI network revealed that MTHFD family genes were coexpressed with several important genes, such as SHMT2 and MTHFR. MTHFD2 and SHMT2 synergistically synthesize tetrahydrofolate in the cell mitochondria. SHMT2 participates in synthesizing 5,10-CH2-THF, and then MTHFD2 utilizes 5,10-CH2-THF to synthesize 10-CHO-THF [22]. Increased SHMT2 expression is associated with poor prognosis in OSCC [23]. Reduced SHMT2 expression has been shown to inhibit OSCC proliferation via altering the expression of cell cycle-related regulators [24]. It was found that MTHFD is irreversibly converted to 5-MeTHF in the intracellular folate metabolism and methionine-homocysteine cycle by the critical enzyme MTHFR [25]. A meta-analysis revealed a statistically significant connection between MTHFR gene polymorphisms and the risk of developing OSCC [26]. This indicates that MTHFD family genes promote cancer progression through interacting with other metabolizing enzymes.

The most significant biological processes enriched by MTHFD family genes-strongly correlated genes included regulation of cell cycle and DNA replication in OSCC. In non-small-cell lung cancer (NSCLC), downregulation of MTHFD2 expression suppresses the expression of cell cycle-related genes, such as CCNA2, MCM7, and SKP2 [27]. MTHFD1 controls the allocation of folate active single-carbon units between the folate-dependent de novo thymidylate and homocysteine remethylation pathways. When MTHFD1 activity is impaired, the homocysteine remethylation and de novo thymidylate production would be impaired, resulting in genomic instability [28].

Previous studies reported that tumor immune infiltration and tumor microenvironment might be involved in tumorigenesis and response to immunotherapy [29, 30]. We found that MTHFD family gene expression was significantly associated with immune cell infiltration, such as Treg and Th17 cells. Increased Treg/Th17 ratio indicated a poor prognosis of OSCC patients. Furthermore, decreasing Treg/Th17 cell levels improved the efficacy of tumor immunotherapy in OSCC patients [31]. These results indicated that MTHFD family genes might play an important role in modulating the tumor immune microenvironment.

We further analyzed the correlations between MTHFD family gene expression and immune-related genes. Our study showed that MTHFD family genes were significantly correlated with several immune inhibitory genes such as CD274 and CTLA-4 and several immune-stimulatory genes such as CXCL12, CXCR4, and TMIGD2. PD-L1 and CTLA-4 expression was significantly increased during nimotuzumab therapy, and their expression prior to nimotuzumab therapy was negatively correlated with overall survival in OSCC [32]. MTHFD2 was overexpressed in various cancer cells and further elevated by IFN-*γ*, enhancing PD-L1(CD274) production at basal and IFN-induced levels. MTHFD2 enhanced PD-L1 transcription by driving the folate cycle to maintain cellular acylation of UDP-GlcNAc and cMYCO-GlcN [33]. These studies indicated that MTHFD family genes might promote OSCC progression via regulating immune inhibitory genes. Cancer-associated fibroblasts differentiated monocytes into M2 macrophages by regulating the CXCL12/CXCR4 pathway, and then M2 macrophages could transform OSCC cells into cancer stem cell-like cells, which enhanced OSCC proliferation [34]. TMIGD2 expression was decreased in OSCC and dysplasia tissues as compared with normal tissues. Moreover, TMIGD2 could serve as an independent indicator of poor prognosis in OSCC [35]. MTHFD family genes may promote OSCC progression by reducing the expression of these immune-stimulatory genes. Immune-related genes and immune infiltrating cells are closely related to OSCC pathogenesis. Thus, further studies are needed to elucidate their roles in OSCC. A deeper understanding of MTHFD family genes in this context could enhance the effectiveness of immunotherapy.

There were some potential limitations to this study. Firstly, the functional profiles and molecular mechanism of MTHFD family genes in OSCC development remain unknown and require further exploration in vitro or in vivo experiments. Secondly, the prognostic values of MTHFD family genes in OSCC should be further validated by the multicenter data to facilitate its implementation in the clinic. Finally, the association between MTHFD family genes and immunity requires further research.

## 5. Conclusion

Our study verified the value of MTHFD family genes in the diagnosis and predicting prognosis of OSCC. MTHFD family genes were overexpressed in OSCC and adversely correlated with worse survival prognosis. Further analysis showed that the correlated genes of MTHFD family genes were enriched in several tumor progression-related pathways such as Aurora B pathway, PLK1 pathway, and cell cycle pathway. Moreover, MTHFD family gene overexpression might also be associated with the abnormal immune microenvironment. This study expands the understanding of MTHFD family gene function in OSCC and suggests that MTHFD family genes might be potential biomarkers and therapeutic targets for OSCC. Further functional experiments and molecular mechanisms are still needed to validate our findings and promote the clinical application.

## Figures and Tables

**Table 1:** The correlations between MTHFD family genes and clinical characteristics by logistic regression analysis.

Characteristics	MTHFD1			MTHFD1L			MTHFD2			MTHFD2L		
	Total (*N*)	Odds Ratio (OR)	*P* value	Total (*N*)	Odds Ratio (OR)	*P* value	Total (*N*)	Odds Ratio (OR)	*P* value	Total (*N*)	Odds Ratio (OR)	*P* value
T stage (T3 and T4 versus T1 and T2)	319	1.309 (0.834–2.061)	0.243	319	1.282 (0.817–2.019)	0.281	319	1.733 (1.101–2.743)	**0.018**	319	1.474 (0.938–2.326)	0.093
N stage (N1, N2, and N3 versus N0)	315	1.456 (0.934–2.276)	0.098	315	1.123 (0.721–1.750)	0.609	315	1.378 (0.884–2.152)	0.157	315	1.119 (0.718–1.744)	0.619
M stage (M1 versus M0)	312	0.975 (0.038–24.791)	0.985	312	82002730.329 (0.000-NA)	0.996	312	1.026 (0.040–26.104)	0.985	312	0.000 (NA-2380550791035)	0.996
Clinical stage (Stage III and Stage IV versus Stage II and Stage I)	319	1.491 (0.914–2.446)	0.111	319	1.215 (0.746–1.985)	0.435	319	1.741 (1.064–2.872)	**0.028**	319	1.270 (0.780–2.078)	0.337
Radiation therapy (Yes versus No)	295	0.678 (0.423–1.084)	0.105	295	1.185 (0.742–1.894)	0.477	295	1.229 (0.770–1.967)	0.388	295	1.135 (0.711–1.816)	0.595
Primary therapy outcome (PD and SD versus CR and PR)	278	0.911 (0.460–1.798)	0.787	278	0.911 (0.460–1.798)	0.787	278	1.784 (0.900–3.637)	0.102	278	0.754 (0.377–1.485)	0.416
Gender (Male versus Female)	329	1.009 (0.632–1.611)	0.971	329	0.900 (0.563–1.437)	0.660	329	1.505 (0.942–2.418)	0.089	329	1.690 (1.056–2.723)	**0.030**
Race (Asian and Black or African American versus White)	318	1.363 (0.641–2.962)	0.423	318	1.826 (0.852–4.097)	0.129	318	1.586 (0.744–3.494)	0.238	318	1.459 (0.684–3.214)	0.334
Age (>60 versus ≤60)	328	0.951 (0.616–1.467)	0.820	328	0.999 (0.647–1.541)	0.995	328	1.340 (0.868–2.073)	0.187	328	0.611 (0.394–0.944)	**0.027**
Histologic grade (G3 and G4 versus G1 and G2)	321	1.126 (0.661–1.925)	0.662	321	1.848 (1.077–3.223)	**0.027**	321	1.566 (0.917–2.701)	0.102	321	1.046 (0.613–1.785)	0.869
Smoker (Yes versus No)	323	0.977 (0.597–1.599)	0.927	323	1.041 (0.636–1.704)	0.874	323	1.147 (0.701–1.879)	0.586	323	1.041 (0.636–1.704)	0.874
Alcohol history (Yes versus No)	321	1.079 (0.676–1.722)	0.751	321	1.119 (0.702–1.787)	0.636	321	1.352 (0.848–2.164)	0.206	321	1.490 (0.933–2.392)	0.096
Lymphovascular invasion (Yes versus No)	239	1.032 (0.597–1.786)	0.911	239	0.778 (0.449–1.344)	0.368	239	1.273 (0.736–2.213)	0.389	239	0.923 (0.533–1.595)	0.774
Lymph node neck dissection (No versus Yes)	327	1.143 (0.608–2.161)	0.678	327	1.143 (0.608–2.161)	0.678	327	1.045 (0.556–1.971)	0.890	327	1.588 (0.842–3.055)	0.157

The bold values means p value < 0.05, and the other unbold values mean p value > 0.05.

## Data Availability

The datasets used and/or analyzed in this study are available from the corresponding author upon reasonable request.
